# Supplementary material for: The causal relationship between the human gut microbiota and pyogenic arthritis: a Mendelian randomization study
Source: Front Cell Infect Microbiol. 2024 Nov 26;14:1452480. doi: 10.3389/fcimb.2024.1452480 (PMC11629706; doi:10.3389/fcimb.2024.1452480)
Supplement: Supplementary file 1 [file DataSheet1.zip › mendelian test/ebi-a-GCST90027672.csv_leave_one_out.pdf]

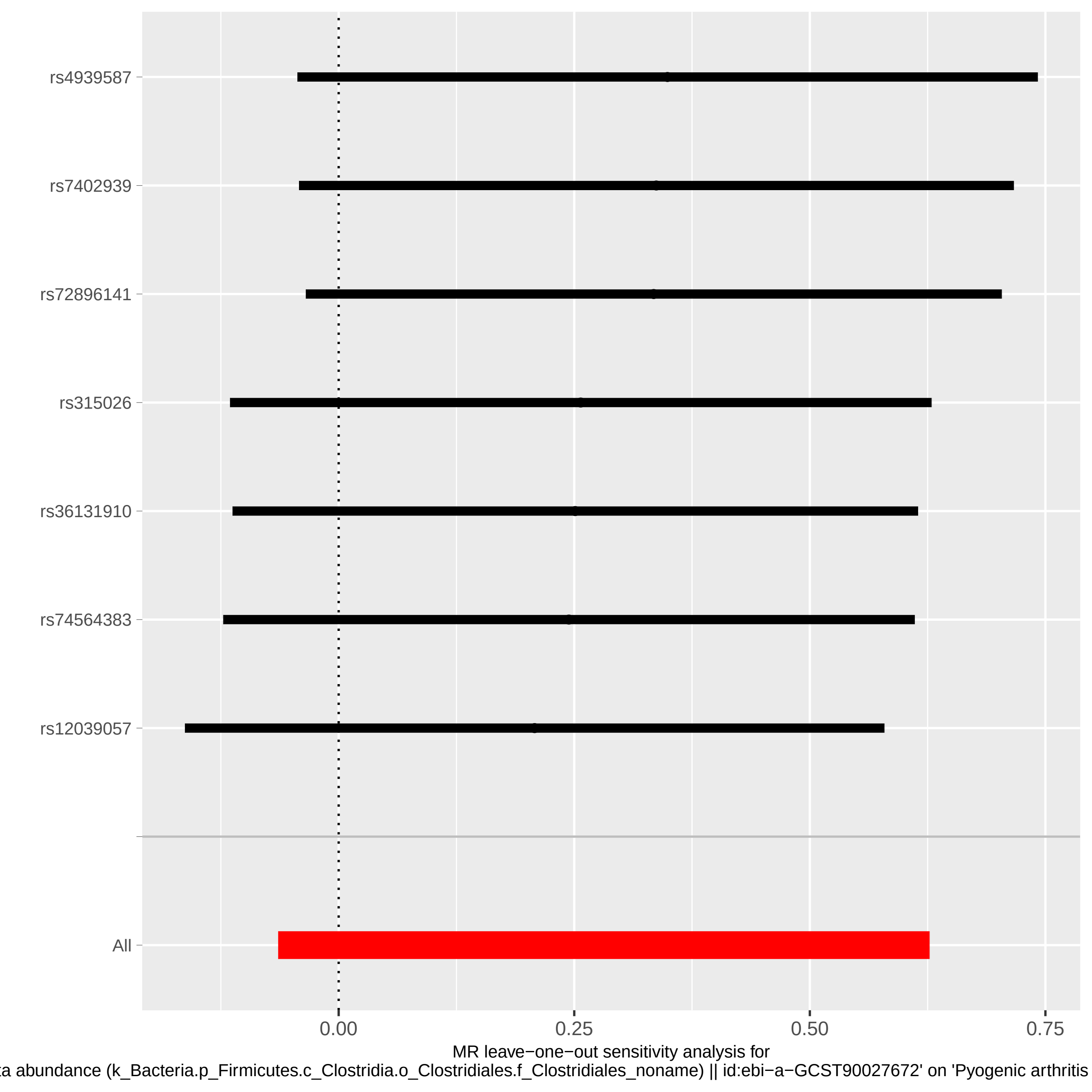

rs4939587
rs7402939
rs72896141
rs315026
rs36131910
rs74564383
rs12039057
All
0.00
0.25
0.50
0.75
MR leave−one−out sensitivity analysis for
ta abundance (k_Bacteria.p_Firmicutes.c_Clostridia.o_Clostridiales.f_Clostridiales_noname) || id:ebi−a−GCST90027672' on 'Pyogenic arthritis
